# Confirmatory factor analysis of competing PANSS negative symptom models: data from OPTiMiSE first-episode schizophrenia study

**DOI:** 10.1192/bjo.2025.10922

**Published:** 2025-12-12

**Authors:** Arsime Demjaha, Daniel Stahl, Celso Arango, Birte Glenthøj, Roberto Rodriguez-Jimenez, Covadonga Martinez Díaz-Caneja, Lone Baandrup, Bjørn Ebdrup, Maria Paz Garcia-Portlla, Maria Diaz-Marsa, Inge Winter-van Rossum, Rene Kahn, Paola Dazzan, Philip McGuire

**Affiliations:** Department of Psychosis Studies, Institute of Psychiatry, Psychology & Neuroscience, King’s College London, London, UK; Department of Biostatistics and Health Informatics, Institute of Psychiatry, Psychology & Neuroscience, King’s College London, London, UK; Department of Child and Adolescent Psychiatry, Institute of Psychiatry and Mental Health, Gregorio Marañón General University Hospital, Health Research Institute Gregorio Marañón (IiSGM), Biomedical Research Networking Centre in Mental Health (CIBERSAM), School of Medicine, Complutense University of Madrid, Madrid, Spain; Center for Neuropsychiatric Schizophrenia Research (CNSR) and Center for Clinical Intervention and Neuropsychiatric Schizophrenia Research (CINS), Mental Health Centre Glostrup, Copenhagen University Hospital, Copenhagen, Denmark; Department of Clinical Medicine, Faculty of Health and Medical Sciences, University of Copenhagen, Copenhagen, Denmark; Complutense University of Madrid, Madrid, Spain; Department of Psychiatry, 12 de Octubre University Hospital Health Research Institute, Madrid, Spain; Biomedical Research Networking Centre in Mental Health – Carlos III Health Institute (CIBERSAM-ISCIII), Madrid, Spain; Center for Neuropsychiatric Schizophrenia Research (CNSR), Mental Health Center Glostrup, Copenhagen University Hospital, Mental Health Services CPH, Copenhagen, Denmark; Faculty of Health and Medical Sciences, Department of Clinical Medicine, University of Copenhagen, Copenhagen, Denmark; Department of Psychiatry, Brain Center Rudolf Magnus, Utrecht, The Netherlands; Department of Psychiatry, Icahn School of Medicine at Mount Sinai, New York, New York, USA; Department of Psychosis Studies, Institute of Psychiatry, Psychology and Neuroscience, King’s College London, London, UK; Department of Psychiatry, University of Oxford, Oxford, UK

**Keywords:** Negative symptoms, schizophrenia, first-episode psychosis, confirmatory factor analysis, negative symptom dimensions

## Abstract

**Background:**

The negative symptoms of psychosis are heterogeneous, which complicates efforts to understand their pathophysiology and develop effective treatments. Factor analytic studies of the Positive and Negative Syndrome Scale (PANSS) have reported two factorial negative symptom models, expressive deficit and social amotivation, albeit with different compositions. Although models derived from other assessment scales have been directly compared, no study has previously applied this approach to PANSS.

**Aims:**

Our objectives were to (a) to establish which negative PANSS-derived factorial model provided the best fit to our data, (b) test its stability and (c) determine its clinical and demographic correlates.

**Method:**

A cohort of medication naive or minimally treated patients with first-episode schizophrenia (*n* = 446) were assessed using the PANSS scale before and 4 weeks after amisulpride treatment. Confirmatory factor analysis was performed to test five PANSS models. Hierarchical multiple regression was conducted to examine the associations between identified dimensions and clinical and demographic variables.

**Results:**

A nine-item PANSS model comprising social amotivation and expressive deficit dimensions outperformed the other models: comparative fit index = 0.98, goodness of fit index = 0.97, Tucker–Lewis index = 0.97, root mean square error of approximation = 0.06 (CI 90%: 0.04–0.08), Bayesian information criterion = 191.9, Akaike information criterion = 101.7. At baseline, the social amotivation dimension was associated with more severe depression whereas the expressive deficit dimension was associated with younger age. Both dimensions at baseline were associated with poor functioning, but expressive deficit to a lesser extent.

**Conclusions:**

A nine-item PANSS model incorporating social amotivation and expressive deficit dimensions appeared to best reflect the underlying structure of negative symptoms in our sample.

Negative symptoms in schizophrenia are difficult to treat and therefore associated with unfavourable clinical and functional outcomes. Their precise phenomenological delineation is crucial for identification of reliable biomarkers and development of effective treatments.^1,2^ Although research has focused on defining the most accurate set of negative symptoms to be included in clinical and research settings, a consensus remains elusive, hindering clinical outcomes. Depending on the assessment scale, studies have identified 2–5 dimensions, at different stage of psychosis.^3–5^ Peralta & Cuesta (1995)^3^ were among the first to test models derived from the Scale for the Assessment of Negative Symptoms (SANS)^6^, using confirmatory factor analysis (CFA), and concluded that a five-factor model was the optimal model. Strauss and colleagues (2018)^7^ investigated several factor models applied to data acquired using the SANS, the Brief Negative Symptom Scale^8^ and the Clinical Assessment Interview for Negative Symptoms (CAINS).^9^ They demonstrated that a pentagonal model comprising blunted affect, anhedonia, alogia, asociality and avolition outperformed other models, in line with a proposal from the National Institute of Mental Health.^3^ However, to our knowledge, no study has previously compared Positive and Negative Syndrome Scale (PANSS) negative factor models, although PANSS is the most widely used rating scale in psychosis research.^10.11^ In contrast to SANS and CAINS, which segregate a wide range of negative items into specific domains, the PANSS negative subscale consists of a small number of single items and has been criticised for poor definition of negative symptoms.^12,13^ Previously, we have demonstrated that the PANSS negative subscale does not capture the underlying latent parameter according to the Rasch model.^14^ Congruently, factor analytic studies of PANSS data have identified a negative factor containing only five items from its negative subscale: flat affect (N1), emotional withdrawal (N2), poor rapport (N3), passive social withdrawal (N4) and lack of spontaneity (N6), with difficulty in abstract thinking (N5) and stereotyped thinking (N7) no longer regarded as negative symptoms, although a European Psychiatric Association (EPA) Guidance Paper on assessment of negative symptoms has recommended their use in clinical and research settings.^15^ However, factor analytic work has also identified a number of PANSS general subscale items (mannerism and posturing (G5), motor retardation (G7), avolition (G13) and active social avoidance (G16)) loading on the negative symptom factor. Motor retardation (G7) and avolition (G13) are of interest, as alogia and markedly affected speech or mutism, reflecting speech disturbances seen in patients with negative symptoms, are found embedded here. The Negative Symptoms Factor Score or ‘Marder factor’, comprising N1, N2, N3, N4 and N6, plus G7 and G16, has attracted particular attention.^16,17^ This model has been replicated and has been shown to have superior content to the original PANSS negative symptoms scale.^18^ Furthermore, it was reflected in two-dimensional CFA-determined models comprising expressive deficit (N1, N3, N6, G7) and social amotivation (N2,N4,G16) dimensions.^19^ Subsequently, Liemburg and colleagues^20^ identified a model reporting the same social amotivation dimension that also reported an expressive deficit dimension consisting of six items (N1, N3, N6, G5, G7 and G13). The best way to characterise the dimensions of PANSS negative items has thus remained unclear. The studies that established the two-dimensional PANSS models were conducted in patients with chronic illness,^4,20^ and the early psychosis studies included medicated samples;^20,21^ these factors may account for the inconsistent reports. Here, we used a CFA approach to directly compare different negative symptom models in a large sample of medication naive and minimally treated patients with first-episode schizophrenia who subsequently underwent a clinical trial of standardised treatment with a single antipsychotic. Although the EPA recommendations that the negative dimension assessed by PANSS should only include items N1, N2, N3, N4 and N6 represent an important approach to refining the negative symptom assessment, evaluation of existing and established models including controversial PANSS general subscale items is crucial to determining the best-performing bidimensional model, which in turn may contribute to evidence-based assessment of negative symptoms. Thus, our objectives were to (a) comparatively assess empirically and theoretically driven PANSS negative bidimensional models, including the five-item model recommended by EPA, as well as established models incorporating a wider range of items, specifically from the PANSS general subscale, to determine which model provided the best fit to our data; (b) investigate whether the structure of the superior model held following standardised treatment; and (c) investigate the validity of this model in relation to external variables.

## Method

Participants were 18–40 years old; met DSM-IV criteria for schizophrenia or schizophreniform or schizoaffective disorder, as defined by the Mini International Neuropsychiatric Interview;^22^ and had been recruited into OPTiMiSE (Optimization of Treatment and Management of Schizophrenia in Europe), a large multicentre trial involving 4 weeks of open-label treatment with amisulpride (www.optimisetrial.eu; EudraCT number: 2010-020185-19; clinicaltrials.gov identifier: NCT01248195). For inclusion, participants were required either to be medication-naive or to have received antipsychotic medications for less than 2 weeks in the previous year or less than 6 months in their lifetime. Exclusion criteria were an interval between onset of psychosis and study entry of more than 2 years; a need for coercive clinical care, representation by a legal guardian or both; being in legal custody; or pregnancy. For full details, see Kahn et al.^23^

### Ethics

All study participants provided written informed consent before taking part. Ethical approval was obtained from the East Research Committee (approval number: 10/H1102/8).

### Clinical assessment

Psychopathology of negative symptoms and overall severity of illness were assessed using PANSS^10^ and the Clinical Global Impression scale (CGI). PANSS encompasses 30 items and consists of three distinct subscales: the positive subscale (seven items), negative subscale (seven items) and general subscale (16 items). The Calgary Depression Scale for Schizophrenia (CDSS)^24^ was used for assessment of depressive symptoms. Level of functioning was measured with the Personal and Social Performance scale.^25^

### Statistical analyses

#### Confirmatory factor analysis

CFA was performed using AMOS version 24 to test seven PANSS bidimensional models of negative symptoms comprising expressive deficit and social amotivation: the nine-item model (Liemburg factor),^4,20^ the three eight-item models based on findings of exploratory factor analyses,^20^ the seven-item model (negative symptoms factor score/Marder factor)^16,18^ and the five-item model adhering to EPA guidance.^15^ CFA is able to accept or reject different models based on associations between unobservable constructs (factors) and observed variables (symptoms). Furthermore, it allows for direct comparison of competing models and for the model to be modified to improve its goodness of fit (GOF), by yielding modification indices.^3,26^ While CFA uses the predetermined set of symptoms, exploratory factor analysis considers the whole scale.

As the data did not show any significant tendency to non-normality (kurtosis < 2; skewness < 2), we used the maximum likelihood method.^33^ Multiple indices were used to measure the GOF: the chi-squared (*χ* ^2^) and standardised root mean square residual (SRMR), which indicate ‘an absolute fit’; the Tucker–Lewis index, which measures ‘relative fit’; and the root mean square error of approximation (RMSEA) and comparative fit index (CFI), which are non-centrality-based indices. Acceptable fit is indicated by TFI and TLI values > 0.9, RMSEA < 0.10, SRMR < 0.08, and normed *χ* ^2^ (the *χ* ^2^ value divided by the degree of freedom, to account for sample size) < 5.^27,28^ To improve the fit, residuals that were highly correlated were introduced into the model.

Model comparison was conducted using the Akaike information criterion (AIC) and Schwarz’s Bayesian information criterion (BIC), which permit comparison of different non-nested latent variables models. Information criteria measure the GOF, including a forfeit for the number of parameters estimated. The best performing model is the model that has the lowest AIC and BIC, and a model that has ten or more units difference compared with the best model is regarded as a weaker or inadequate model.^29^ We performed structural equation model assessments to compare the fit of the predicted covariance matrix relative to the observed covariance matrix. AIC and BIC model selection can only be used to compare non-nested models with the same set of observed variables and the same number of observations. We therefore used the full set of observed variables in the analyses and fixed the paths that were not of interest to zero.

#### Hierarchical multiple regression

Linear or logistic regressions as appropriate were employed to examine the associations of the social amotivation (total score for PANSS items N2, N4 and G16) and expressive deficit (total score for PANSS items N1, N3, N6, G5, G7 and G13) dimensions of the best fitting model with clinical and demographic parameters that have been associated with negative symptoms in previous literature: depressive symptoms, age, social functioning, education years and living status.^4,5,30,31^ Total scores of the two dimensions at a baseline were entered as independent variables, whereas demographic and clinical variables were included as dependent variables (model 1), while covarying for gender and PANSS positive symptoms total score (model 2). Multicollinearity was assessed using the variance inflation factor (VIF) and tolerance statistics. All statistical analyses were performed with IBM SPSS Statistics version 22 for Windows (IBM Corp, Armonk, NY, USA; see https://www.ibm.com/products/spss-statistics).

Paired-samples *t*-tests were used to examine the change in negative symptoms following treatment with amisulpride.

## Results

### Sample description

Demographic and clinical characteristics are presented in Table 1. Of 446 patients who completed the PANSS at baseline, 368 (83%) were re-assessed using the PANSS, 4 weeks post-treatment with amisulpride (Table 1). There were no significant demographic or clinical differences between the samples of patients assessed at baseline and at follow-up. The average amisulpride dose received during the treatment period was 416.7 mg daily (Table 1).

**Table 1:** Clinical and demographic characteristics of the sample at baseline (pre-treatment) and 4-week (post-treatment) follow-up

	Pre-treatment (*N* = 446)	Post-treatment (*N* = 368)
Age in years, mean [s.d.]	26 [6.0]	25.3 [5.7]
Gender, *N* [%]		
Man	312 [70]	267 [72.6]
Woman	134 [30]	101 [27.4]
Ethnicity, *N* [%]		
White	386 [87]	322 [87.5]
Other	60 [13]	46 [12.5]
Years of education, *mean [s.d.]	12.3 [3.0]	12.4 [2.9]
Living status, *N* [%]		
Living independently	83 [19]	72 [20]
Living with assistance	363 [81]	296 [80]
Diagnosis, *N* [%]		
Schizophrenia	229 [51]	197 [52]
Schizophreniform disorder	188 [43]	150 [41]
Schizoaffective disorder	27 [6]	21 [6]
PANSS total mean [s.d.]	78.2 [18.7]	56.3 [17.9]
PANSS negative subscale	19.4 [7.1]	16.1 [6.6]
PANSS positive subscale	20.2 [5.5]	12.9 [5.1]
PANSS general subscale	38.6 [9.8]	27.3 [8.5]
CGI severity	4.5 [0.9]	4.4 [1.2]

CGI, Clinical Global Impression scale; PANSS, Positive and Negative Syndrome Scale.

### Model comparisons

The nine-item bidimensional model comprising social amotivation (N2, N4, G16) and expressive deficit (N1, N3, N6, G5, G7, G13) dimensions showed a considerably lower AIC and BIC than the other four models and superior model fit indices; thus, it provided the best fit for the data (Table 2).

**Table 2:** Comparison of PANSS negative symptom models at baseline: model fit indices for bidimensional social amotivation/expressive deficit models derived from different combinations of PANSS items

Model	AIC	BIC	RMSEA (90% CI)	TLI	CFI	RMR	GFI
Five-item;^19^ social amotivation (N2, N4), expressive deficit (N1, N3, N6)	494.43	568.235	0.19 (0.17–0.21)	0.673	0.755	0.455	0.809
Seven-item (Marder factor);^20^ social amotivation (N2, N4, G16), expressive deficit (N1, N3, N6, G7)	251.203	252.123	0.13 (0.11–0.15)	0.848	0.894	0.320	0.917
Eight item 1;^25^ social amotivation (N2, N4, G16), expressive deficit (N1, N3, N6, G7, G13)	168.801	254.908	0.09 (0.08–0.12)	0.912	0.942	0.188	0.944
Eight-item 2;^25^ social amotivation (N2, N4, G16), expressive deficit (N1, N3, N6, G5, G7)	224.984	311.091	0.12 (0.11–0.14)	0.864	0.91	0.279	0.926
Eight-item 3;^25^ social amotivation (N2, N4), expressive deficit (N1, N3, N6, G5, G7, G13)	186.558	272.664	0.11 (0.09–0.12)	0.897	0.931	0.242	0.937
Nine-item (Liemburg factor);^25^ social amotivation (N2, N4, G16), expressive deficit (N1, N3, N6, G5, G7, G13)	101.686	191.893	0.06 (0.04–0.08)	0.969	0.98	0.057	0.972

N1, flat Affect; N2, emotional withdrawal; N3, poor rapport; N4, passive social withdrawal; N6, lack of spontaneity; G5, mannerism and posturing; G7, motor retardation; G13, avolition; G16, active social avoidance; AIC, Akaike information criterion; BIC, Schwarz’s Bayesian information criterion; RMSEA, root mean square error of approximation; TLI, Tucker–Lewis index; CFI, comparative fit index; RMR, root mean square residual; GFI, goodness of fit index; PANSS, Positive and Negative Syndrome Scale.

### Nine-item model fit (pre-treatment data)

**Fig. 1:**
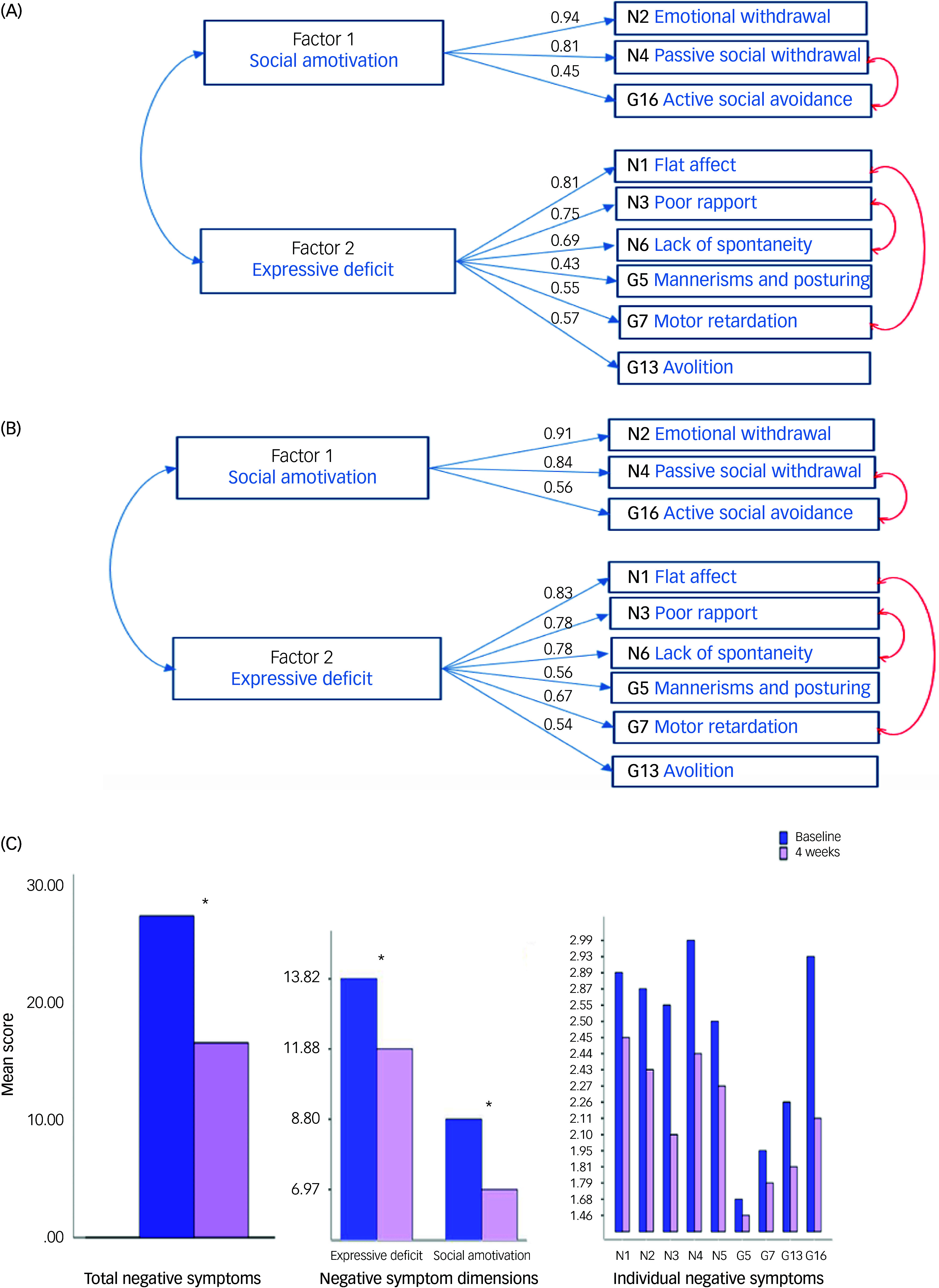
(A) Model fit pre-treatment. (B) Model fit post-treatment. (C) Reduction in negative symptoms following 4 weeks of treatment. Total PANSS (Positive and Negative Syndrome Scale) negative symptoms score (*P* < 0.001). Dimensions: expressive deficit: *P* < 0.001; social amotivation: *P* < 0.001); individual PANSS items: *P* = 0.03 (G5), *P* < 0.001 (all other items). Asterisk indicates significant change from pre- to post-treatment. G5, mannerisms and posturing.

At baseline, the CFA indicated a reasonable fit for a two-factor model of negative symptoms. The GOF indices were CFI = 0.92, goodness of fit index (GFI) = 0.93, TLI = 0.90, RMSEA = 0.10 [90% CI: 0.09–0.13] and SRMR = 0.05. Introduction of residual correlates between N4 (passive/apathetic social withdrawal) and G16 (active social avoidance), N1 (blunted affect) and G7 (motor retardation), and N3 (poor rapport) and N6 (lack of spontaneity), as indicated by high modification indices, significantly improved data fit: CFI = 0.98, GFI = 0.97, TLI = 0.97, RMSEA = 0.05 [90% CI: 0.04–0.08] and SRMR = 0.03 (Fig. 1(A1)). In view of the modest factor loadings on motor retardation (G7) and disturbance of volition (G13), we repeated the analysis excluding these items; however, this produced a much weaker data fit, as indicated by the relatively high RMSEA of 1.5 [90% CI: 0.12–0.19] (CFI = 0.97, GFI = 0.96, TLI = 0.91 and SRMR = 0.04).

### Nine-item model fit (post-treatment data)

When applied to the data collected after 4 weeks of treatment, the CFA produced the following GOF indices: CFI = 0.96, GFI = 0.95, TLI = 0.95, RMSEA = 0.08 [90% CI: 0.06–0.10] and SRMR = 0.02. Introduction of residual correlates in the model yielded a better fit (CFI = 0.98, GFI = 0.97, TLI = 0.97, RMSEA = 0.06 [90% CI: 0.05–0.09] and SRMR = 0.03), indicating very similar data fit (Fig. 1(A1)). The social amotivation dimension was evident in 85% of patients at baseline and in 71% at follow-up. Similarly, the expressive deficit dimension was present in 86% of patients at baseline and 79% at follow up. The PANSS items with the highest scores were passive social withdrawal (N4) and active social avoidance (G16) (Fig. 1(C1)).

The PANSS negative total symptom score significantly decreased following treatment with amisulpride: pre-treatment mean = 22.4 (s.d. = 8.4), post-treatment mean = 18.9 (s.d. = 7.7) (*t* = 10.1, d.f. = 367, *P* > 0.001). The effect size for this change was moderate (Cohen’s *d* = 0.59 [95% CI: 0.4–0.6]). Similarly, social amotivation and expressive deficit dimensional mean scores were significantly lower at 4 weeks. The mean social amotivation scores were 8.7 (s.d. = 3.7) pre-treatment and 7.0 (s.d. = 3.3) post-treatment, with an effect size of 0.6 [95% CI: 0.4–0.9]. The mean expressive deficit scores were 13.9 (s.d. = 5.7) pre-treatment and 11.9 (s.d. = 5.0) post-treatment, with an effect size of 0.4 [95% CI: 0.3–0.5].

### Associations with demographic and clinical parameters

Higher social amotivation scores were significantly associated with more depressive symptoms (CDSS), whereas higher expressive deficit scores were significantly related to younger age at onset.

Both dimensions predicted worse functioning (Personal and Social Performance scale total score), but the expressive deficit dimension to a lesser degree than the social amotivation dimension. The relationships remained significant after covarying for gender and PANSS positive symptoms total score (Table 3). Logistic regression analyses revealed no significant associations between social amotivation or expressive deficit scores and living status (odds ratios of 0.98 (*P* = 0.59) and 0.99 (*P* = 0.91), respectively. The results remained similar following adjustment for gender and PANSS positive symptoms total score. No multicollinearity was observed among predictors. All VIF values were below 1.28 (VIF range: 1.007–1.280), and all tolerance values exceeded 0.8.

**Table 3:** Linear regression models for depressive symptoms, age and social functioning for two negative symptom dimensions

		CDSS				Age				PSP				Education Level			
		*β*	*t*	*P*	*R* ^2^	*β*	*t*	*P*	*R* ^2^*	β	t	*P*	*R* ^2^*	*β*	*t*	*P*	*R* ^2^*
Model 1	Social amotivation	0.296	6.33	<0.001	0.086	0.021	0.45	0.65	−0.002	−0.321	−6.21	<001	−0.100	−0.021	−0.424	0.672	−0.002
	Expressive deficit	0.060	1.23	0.218	0.001	−0.156	−3.33	<0.001	0.022	−0.138	−2.55	0.01	0.16	0.036	0.737	0.462	−0.001
Model 2^a^	Social amotivation	0.292	5.87	<0.001	0.085	0.037	0.736	0.462	0.015	−0.327	−5.91	<0.001	0.099	−0.018	−0.360	0.719	0.013
	Expressive deficit	0.036	0.713	0.476	0.010	−0.157	−3.28	<0.001	0.037	−0.122	−2.21	0.03	0.019	0.041	0.828	0.408	0.014

CDSS, Calgary Depression Rating Scale for Schizophrenia; PSP, Personal and Social Performance Scale.

a. Model 2 was adjusted for gender and PANSS (Positive and Negative Syndrome Scale) positive symptoms total score.

## Discussion

To our knowledge, this is the first study to examine different PANSS negative symptom models to determine which symptom model may best conceptualise the negative symptom construct when this assessment scale is used. By applying CFA in a large sample of medication naive or minimally treated patients with first-episode schizophrenia, we found that a nine-item model (Liemburg factor)^4,20^ incorporating social amotivation and expressive deficit dimensions had the best fit to our PANSS data. We further demonstrated that this model appeared to be relatively independent of both antipsychotic treatment and overall negative symptom severity. Finally, the correlation analyses revealed some unique correlates of the dimensions identified, which to an extent suggests the external validity of this model.

### Best-fitting PANSS negative symptom model

One of the main findings from the present study is that a nine-item two-dimensional model provided the best fit for negative symptoms rated using the PANSS. Our findings extend results from previous CFA studies that described an identical nine-item model incorporating the social amotivation and expressive deficit dimensions but included medicated patients with either chronic or early-phase schizophrenia and often analysed non-homogeneous samples.^4,20,32^ The social amotivation dimension comprised emotional withdrawal (N2), passive social withdrawal (N4) and active social avoidance (G16), which are related to emotional and/or motivational deficits and thus reflect the ‘loss of motivation or interest’ aspect of negative symptoms.^4^ The expressive deficit dimension included flat affect (N1), poor rapport (N3), lack of spontaneity (N6), mannerism and posturing (G5), motor retardation (G7) and avolition (G13), which are pertinent to ‘loss of initiative’ and reflect language and affect disturbances. Items such as active social avoidance and flat affect could be related to high doses of dopamine-blocking antipsychotics; however, as our sample comprised generally untreated patients, the results are unlikely to be related to effects of previous treatment. Moreover, these items have consistently integrated with other negative symptoms in a myriad of studies,^20^ and in our previous work, inclusion of these nine negative symptom items yielded robust neuroimaging correlates involving orbitofrontal and left superior temporal gyrus, lending further support to this model.^33^ In concordance with previous reports, we found that items N2–N4 and N6 showed the highest loadings, whereas the general subscale items were low-loading items.

**Fig. 2:**
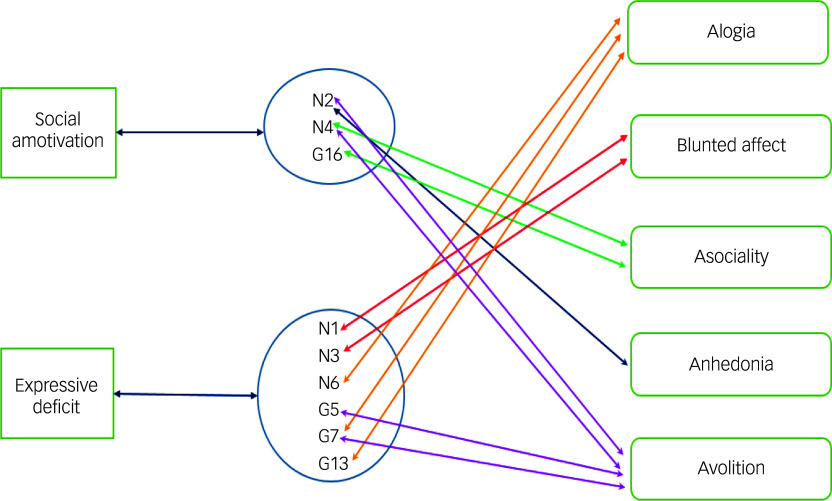
Linking PANSS negative symptoms to five NIMH (SANS)-agreed domains. N1, flat affect; N2, emotional withdrawal; N3, poor rapport; N4, passive social withdrawal; N6, lack of spontaneity; G5, mannerisms and posturing; G7, motor retardation; G13, avolition; G16, active social avoidance. PANSS, Positive and Negative Syndrome Scale; NIMH, National Institute of Mental Health; SANS, Scale for the Assessment of Negative Symptoms.

More generally, our findings confirm a two-dimensional model of negative symptoms^21^ and replicate results from previous studies that used PANSS and also SANS data.^4,20,32^ However, our findings were not in line with the pentagonal negative symptom dimensional model recently proposed by Strauss and colleagues.^5,26^ They challenged the bimodal representation of negative symptoms in DSM-5 but acknowledged that their data were from patients with chronic schizophrenia. In addition, the SANS/CAINS/Brief Negative Symptom Scale pentagonal model is based on a relatively wide range of negative symptoms that are structured *a priori* in several categories. By contrast, the PANSS groups singular negative symptom items under the umbrella of its negative subscale. Owing to this relatively small number of items, even when general subscale items are included, the PANSS does not lend itself to the more complex structures seen when using the SANS or CAINS. Nevertheless, although the models are structurally different, the nine items in the PANSS model can be linked adequately to the items from SANS/CAINS models, as illustrated in Fig. 2.

### Stability of the nine-item model in relation to antipsychotic treatment and severity of symptoms

Our data indicate that the social amotivation and expressive deficit dimensions within the nine-item model are relatively stable and not related to negative symptom severity or treatment. After 4 weeks of treatment, the negative symptoms had a very similar structure to the baseline, with the composition of dimensions remaining unchanged (Fig. 1 (B1)). This was consistent with previous evidence that the severity of negative symptoms has little effect on the factorial structure.^26^ To our knowledge, no previous studies have examined the stability of negative symptom constructs specifically in relation to antipsychotic medication; however, our findings need replication in studies involving longer periods of treatment.

Both the social amotivation and expressive deficit dimensions were present in a large majority of the patients and in similar proportions at the two time points. There was a significant decrease in the mean dimension scores following 4 weeks of treatment with amisulpride, with a 14% reduction in negative symptom severity for expressive deficit and a 17% reduction for social amotivation. Ergul and colleagues,^30^ in their follow-up study, observed reduction in the severity of negative symptoms at 3 months after initiating antipsychotic treatment. A decrease in negative symptom severity after only 4 weeks of treatment in our study suggests that these symptoms may be more responsive to antipsychotic medications in the early phase of psychosis. However, it is also possible that the effect was related to the concurrent reduction in positive psychotic symptoms in our sample.^30,35^

### Differential relationships of social amotivation and expressive deficit dimensions

Our results established distinct correlates of the social amotivation and expressive deficit dimensions. The social amotivation dimension was associated with more depressive symptoms. Generally, studies have reported associations of depression with negative symptoms,^36,37^ even in the prodromal stage of psychotic illness,^38^ although not all studies have found this association.^39^ The association between the social amotivation dimension and depression here, which has been also documented in two previous studies,^4,20^ and the absence of a relationship between depression and the expressive deficit dimension suggest that the previously reported link between depression and negative symptoms may be driven by the social amotivation dimension. Although our correlation analyses demonstrated robust association between social amotivation and CDSS scores, the potential overlap with depressive symptoms merits consideration, as these may mimic or amplify some aspects of social amotivation, particularly the social withdrawal item. This may have implications for negative symptom assessment in clinical settings, particularly in early psychosis, in which affective symptoms are more prominent. Therefore, the absence of association of CDSS scores with expressive deficit in our study indicates that expressive deficits may represent a more distinct negative symptom domain. Future research employing longer-term longitudinal designs should focus on further disentanglement of these overlapping symptoms to investigate the chronic stability and distinctiveness of the social amotivation dimension. In the present study, the expressive deficit dimension was associated with earlier age at onset, consistent with data from previous studies.^30^ This association, together with an association with neurocognitive deficits,^30^ suggests that this domain may have a neurodevelopmental aetiology. The observed association of the social amotivation dimension with poor functioning was in line with evidence of a robust relationship between this dimension and social dysfunction. This was not surprising, given that the social amotivation dimension comprised items relating to active and passive social and emotional withdrawal and/or avoidance. In the present study, we also found that the expressive deficit dimension was related to poorer social functioning, but to a lesser extent than the social amotivation dimension. Previous studies have found social and functional impairments to be more closely associated with the social amotivation than the expressive deficit dimension,^30,34^ but a significant correlation between expressive deficit and functional impairment has also been reported.^40^ In contrast with previous research,^4^ no association between social amotivation or expressive deficit and living situation was observed here.

### Strengths and methodological consideration

Most previous studies of negative symptom dimensions have involved medicated patients, mostly in chronic populations involving non-homogeneous samples at various stages of their illness, often not distinguishing primary from secondary negative symptoms.^26^ A key strength of our study was that it examined negative symptoms in a large cohort of antipsychotic-naive or minimally treated first-episode schizophrenia patients, involving a strict treatment protocol;^23^ this minimised the potentially confounding effects of illness duration and previous treatment, allowing more accurate characterisation of primary negative symptoms. This may have important clinical implications for early intervention treatment strategies and the development of potential biomarkers. A further strength was the use of CFA, which is superior to other factor analytical methods, as it permits robust testing and direct statistical comparisons between different models and generates modification indices, providing a means for improving the model.^5^ Finally, in light of the inherent limitations of the PANSS negative subscale, our data provide further support for more adequate identification of negative symptoms when this scale is used. One potential limitation is the relatively brief duration of the treatment period. On the other hand, treatment was standardised and involved a single antipsychotic at similar doses across patients. We used a small number of clinical and demographic factors to assess to an extent the external validity of the nine-item model, as our study was done in the context of a clinical trial; we acknowledge that associations with cognitive, genetic and neuroimaging indices should be investigated in future research to establish robustly the external validity of the model. Further, we acknowledge the impact of substance misuse on negative symptoms. As only 15% of participants reported current or recent drug use at baseline, it is unlikely that our results were confounded by substance misuse. Our sample was predominantly male and of White ethnicity, which may limit the generalisability of our findings in light of reports that negative symptoms are more prevalent in males^40^ and given the potential effect of ethnicity on rating of negative symptoms, specifically emotional or social withdrawal. Future research should aim to validate this model in more culturally diverse and clinically complex populations.

A nine-item bidimensional model (Liemburg Factor) appears to best reflect the underlying structure of negative symptoms. Our overall findings suggest that this model could provide a more reliable framework in future psychosis research when using PANSS collected data. While this model may enhance the precision of negative symptom assessment and potentially improve clinical outcomes through more accurate treatment stratification, further validation is warranted before it can be considered optimal for assessment of negative symptoms.

## Data Availability

The data that support the findings of this study are available from the corresponding author, A.D., upon reasonable request.
